# Contributions of nurse specialists in the allergy and immunology service and patient care

**DOI:** 10.5339/qmj.2022.fqac.7

**Published:** 2022-04-13

**Authors:** Yaldez Ibrahim, Slim Bin Naji, Hassan Mobayed, Maryam Al-Nesf

**Affiliations:** ^1^Allergy and Immunology Division, Department of Medicine, Hamad Medical Corporation, Doha, Qatar E-mail: YIbrahim2@hamad.qa

**Keywords:** allergy and immunology, nurse specialists, patient care

## Abstract

Specialist nurses have a crucial role within the allergy and immunology specialty that reflects their competence in an increasingly complex area of work and ability to use advanced diagnostic testing and therapeutic modalities. A specialist nurse in Qatar follows the local Hamad Medical Cooperation (HMC) guidelines and the competency published by the British Society for Allergy and Clinical Immunology (BSACI).

Specialist Allergy Nurses

Learning domain: Learn and review the written standard operating procedure (SOP) and protocols for all diagnostic and therapeutic allergy procedures, including skin prick testing (inhaled, food, and others), intradermal testing, skin patch testing, penicillin testing, food and drug challenges, different desensitization protocols, and biologics and other medication administration. In addition, learn the protocol for immunotherapy administration and maintain an updated knowledge of trends and developments in the field by reading relevant articles, journals, and related material and attending seminars and conferences, as needed.

Maintain a safe work environment: Perform all the above procedures and medication administration with a high grade of competency under physician supervision. Apply quality measures to ensure safety. Furthermore, perform stocktaking by regularly checking procedure requirements, availability of items and consumables, date of expiry of items, and medications. Monitor the patients closely during drug and food allergy challenge procedures and record and report patients’ vitals and reactions to treating physicians. Ensure that a patient stays the required observation time after injection(s).

General care: Assess patient needs and suggest solutions to patient care problems. Provide education and resources for the patient and family support as needed. React appropriately and on time during patient reaction situations to mitigate early adverse reactions. Maintain patient, employee, and clinic confidentiality.

Specialist Immunology Nurses

• Develop specialist knowledge and experience in immunology.
• Develop nurse-led clinics for administering immunoglobulin replacement therapy safely and effectively based on patients’ preferences (IV vs. SC and in-hospital vs. home programs).
• Coordinate communication between members of the multidisciplinary team to facilitate the appropriate delivery of care.
• Provide education and resources for the patient and family support (insurance issues, resources for vaccination recommendations, concerns regarding traveling and vacations, pregnancy, and any other lifecycle changes).
